# A Case of Stricture of the Urethra, Complicated with Hypertrophy of the Prostate, Stone and Violent Cystitis

**Published:** 1877-10

**Authors:** Shirley Bragg

**Affiliations:** Atlanta, Ga.


### A CASE OF STRICTURE OF THE URETHRA COMPLICATED WITH HYPERTROPHY OF THE PROSTATE, STONE, AND VIOLENT CYSTITIS.

By SHIRLEY BRAGG, M.D., ATLANTA, GA.

On August 24th, shortly after an operation (external urethrotomy) I was called in consultation by my friend, Dr. R., of this city, to see Mr. F., (aged 60) who, to all appearances, was suffering only from a violent attack of cystitis, with a constant desire to urinate, and the unpleasant symptoms accompanying any inflammatory action of the vesical neck.

The Doctor had tried the usual remedies which promise most relief, and, finding his patient no better, urged upon him the necessity of an examination, to which, finally, he consented. Accordingly his diuretic was continued with an enema of starch and tr. opii, forty drops to the ounce, to quiet the constant tenesmus.

The following evening, I determined to examine him carefully, expecting to find some enlargement of the prostate, which, in one of his temperament, is not infrequent. The urethra was first explored, and found to be exceedingly irritable, though normal, so far as the bulb. At this juncture a stricture was perceptible, not, however, of small calibre. After satisfying myself as to this point, the prostate was carefully examined, through the rectum. As anticipated, I found a very great enlargement of that organ. In fact, so far as could be judged from a sense of touch, the whole bladder seemed to be indurated. After consultation, Dr. R. and myself determined to relieve the strictured urethra, not by rapid divulsion, but gently stretching the parts with divulsor. The enema was still kept up, and after passing the night more quietly than had been his custom to do, was seemingly better. He was now kept quiet in bed. Quinia and ferri administered, with a generous diet.

A few days later, finding him passing so little water, and with such difficulty, I determined to introduce the catheter gently, drawing off what residue there was, and washing out the bladder. This was found to give great relief, so much, indeed, that I concluded to continue it daily, using simply warm water, at first, then a solution of bi-borate sodæ. In the mean time beef-wine and iron had been ordered to fortify his system as much as possible. From the first introduction of the catheter, stone was suspected, for, upon removal of this instrument, I could always perceive, as it were, a grating in one particular spot. Taking the condition of the patient into consideration, the length of time he had been affected, it was patent to my mind that further surgical interference was to be avoided if possible. Upon sounding, carefully, the stone was found to occupy, seemingly, the left side. This led me to believe that, perhaps, it was partially incisted; so, with the consent of the patient, I called on my friend, Dr. O., of this city. Our views coincided as to its presence and position. By this time he was rapidly losing strength, though, to outside appearances, his cystitis was better. Looking carefully to everything, only one conclusion could be arrived at; i. e., should we operate with a bare chance, or quietly wait for what a few days would surely bring forth—death?

Explaining to him and his family his critical condition, an operation was submitted to, which, at best, was a speculative chance. He was much emaciated and extremely weak; in fact, stimulants were being administered freely. There being no time to lose, a slight laxative was ordered, to clear the lower bowel, and the operation performed the following morning, in the presence of Drs. Owen, Logan, Roach, Lee, and Messrs. Johnson and Gordon, students, Dr. Lee kindly administering the anæsthetic. Though very feeble, the pulse improved under its influence, and in a few minutes all was ready for the operation. The staff being introduced, I proceeded to do the lateral operation, making my incision into the prostate as small as possible, leaving the rest to dilatation. A few moments more, and my finger glided over the staff into the bladder, giving me control of that organ. I was now somewhat amazed to find no stone, but upon pressure above the pelvis with my disengaged hand my finger readily came in contact with it. Withdrawing the staff and introducing the forceps along my finger as a guide, I slowly extracted a calculous, undoubtedly of uric acid, measuring about $1\frac{1}{4}$ inches in length, and 1 inch in breadth. The bladder was further explored, cleansed, and the patient returned to bed. Hemorrhage was very slight, and, upon arousing from the effects of chloroform, expressed himself as feeling comfortable. A few minutes later, a severe rigor came on, from which, however, he shortly recovered. He was now put on opium and kept perfectly quiet. That night he rested well, but I found him next morning with some fever and a tense bladder. Complained of not being able to get rid of his water, and, to relieve his troubles in this direction, we agreed to draw it. That evening found him much better than was expected, having rested quietly.

Monday morning (the following day) was not so well, though cheerful. Stimulants were ordered. Later, in the evening, he was evidently sinking, and urine had passed freely. The wound, with the exception of a peculiar dryness, was doing well as could have been expected.

The following morning showed plainly that all our efforts had been in vain, and that night he quietly succumbed, not from septicæmia, pyemia, infiltration, or other concomitant diseases, but simply from exhaustion brought on from long suffering, and a shattered constitution.

